# Coexisting Molecular Alterations Increase the Risk of Malignancy in Thyroid Nodules with Copy Number Alterations

**DOI:** 10.3390/cancers14246149

**Published:** 2022-12-13

**Authors:** Mohannad Rajab, Saruchi Bandargal, Marc Philippe Pusztaszeri, Véronique-Isabelle Forest, Sama Alohali, Sabrina Daniela da Silva, Michael Tamilia, Richard J. Payne

**Affiliations:** 1 Department of Otolaryngology—Head and Neck Surgery, Jewish General Hospital, McGill University, 3755 Côte-Sainte-Catherine Road, Montreal, QC H3T 1E2, Canada; 2 Departments of Otolaryngology—Head and Neck Surgery, Royal Victoria Hospital, McGill University, 1001 Decarie Blvd, Montreal, QC H4A 3J1, Canada; 3 Department of Otolaryngology—Head and Neck Surgery, King Faisal Specialist Hospital & Research Center, Al Madinah Al Munawwarah 42523, Saudi Arabia; 4 Faculty of Medicine, McGill University, 845 Rue Sherbrooke O, Montral, QC H3A 0G4, Canada; 5 Department of Pathology, Jewish General Hospital, McGill University, 3755 Côte-Sainte-Catherine Road, Montreal, QC H3T 1E2, Canada; 6 Department of Endocrinology and Metabolism, Jewish General Hospital, McGill University, 3755 Côte-Sainte-Catherine Road, Montreal, QC H3T 1E2, Canada

**Keywords:** copy number alterations, thyroid cancer, molecular testing, thyroid nodule

## Abstract

**Simple Summary:**

Copy number alterations are known to be present in some thyroid tumors; however, their idiosyncratic clinicopathological implications are not yet well elucidated. In this study we reviewed 67 thyroid nodules with positive copy number alterations who underwent surgery. We discovered that most of the thyroid nodules with positive copy number alterations were malignant or non-invasive follicular thyroid neoplasm with papillary-like nuclear features and the presence of coexisting molecular alterations increased the risk of malignancy in these nodules. These findings may affect the decision-making in individuals with copy number alterations positive thyroid nodules.

**Abstract:**

Molecular mutations and alterations play a role in thyroid tumorigenesis. Different alterations are associated with different clinical and pathological characteristics. Copy number alterations (CNAs) are known to be present in some thyroid tumors; however, their idiosyncratic clinicopathological implications are not yet well elucidated. A retrospective chart review was performed to identify patients with CNAs on pre-operative molecular testing results who subsequently underwent surgical treatment between January 2016 and April 2022 at McGill University teaching hospitals. Of the 316 patients with thyroid nodules who opted for molecular testing with ThyroSeqV3 followed by surgery, 67 (21.2%) nodules were positive for CNAs, including 23 Bethesda III, 31 Bethesda IV, 12 Bethesda V and 1 Bethesda VI nodules. On surgical pathology, 29.9% were benign and 70.1% were malignant or non-invasive follicular thyroid neoplasm with papillary-like nuclear features (NIFTP). Among those that were malignant/NIFTP, 17.02% were considered to be aggressive cancers. The presence of other molecular alterations was found to be an independent predictor of malignancy in multivariate analysis (OR = 5.087, 95% C.I. = 1.12–23.04, *p* = 0.035). No unique factor was correlated with aggressiveness; however, CNA-positive thyroid nodules that were associated with high-risk mutations such as *BRAF V600E*, *TP53*, *NTRK1/3* fusion, or *PTEN* mutation with high allele frequency (AF) ended up being aggressive cancers. Most of the CNA-positive thyroid nodules resulted in follicular patterned tumors in 41 (65.2%) cases and oncocytic tumors in 20 (29.9%) cases. This study demonstrates that 70.1% of surgically resected thyroid nodules with CNAs were malignant/NIFTP. Most CNA-positive thyroid nodules were either oncocytic patterned tumors or follicular patterned tumors. Furthermore, CNA-positive thyroid nodules were more likely to be malignant if they were associated with other molecular alterations or mutations.

## 1. Introduction

The management of thyroid nodules can be a challenge to physicians [1]. The risk of malignancy in these nodules regardless of their size is low (<10%) [2,3]. Ultrasound-guided fine-needle aspiration (FNA) in tandem with cytological evaluation is the current gold standard to risk stratify thyroid nodules and triage those who need further treatment. Nonetheless, about 10% to 30% of thyroid nodules after FNA are classified as indeterminate, with risk of malignancy ranging from 5% to 75%, which can lead to suboptimal treatment [4,5,6].

Genetic landscape of thyroid cancer is well characterized with >90% of genetic driver alterations being known. Most of these driver alterations cause dysregulation of the mitogen-activated protein kinase (MAPK) and phosphatidylinositol-3 kinase (PI3K)/AKT signaling pathways which eventually lead to tumorigenesis [7]. The genotype-phenotype correlation between different molecular alterations and morphologic features of thyroid tumors has been explored by multiple studies [8,9]. Currently, molecular testing for thyroid nodules has made significant progress and is able to identify thyroid cancer-related molecular markers which can then be applied clinically for improved decision-making [10]. 

Multiple studies demonstrated the advancement of molecular genetics and testing in thyroid nodules and cancers [11,12,13,14]. This advancement allowed for optimizing the evaluation and management of indeterminate thyroid nodules and thyroid cancers. It allowed for refining the risk of malignancy in Bethesda III and IV nodules and direct the decision between surveillance and diagnostic/therapeutic thyroidectomies. Furthermore, it assisted in providing more personalized treatment for patients with thyroid cancers by helping in deciding the extent of the surgery and identifying targetable molecular alterations in advanced thyroid cancers [10,15]. Some other studies are evaluating the diagnostic and prognostic applications of molecular characterization in thyroid cancers using liquid biopsy [16].

Copy number alterations (CNAs) are defined as a section of DNA that presents variable copy numbers in comparison with a reference genome [17]. The developmental mechanisms of CNAs are obscure; however, CNAs can be either inherited or acquired spontaneously during mitosis through deletions, duplications, segmental duplications, insertions, inversions, or translocations [18]. The effects of these alterations can vary according to the affected part of the genome and its size [19]. In The Cancer Genome Atlas (TGCA) cohort, CNAs were found in 27% of thyroid tumors. The lack of other mutations or fusions in a lot of these tumors suggested the role of CNAs as a driver alteration in thyroid cancer [11]. Furthermore, CNAs can be a promotor for tumor progression by altering gene expression levels of the affected genes [20].

CNA prevalence and associated phenotypical and clinical characteristics in thyroid nodules are nebulous. The aim of our study is to identify the prevalence of CNAs in thyroid nodules that underwent molecular testing followed by surgical resection at McGill university teaching hospitals, characterize their clinicopathological features, review their surgical outcomes, and identify the possible impact of detecting this alteration on patients’ thyroid nodule management.

## 2. Materials and Methods

### 2.1. Study Design and Patient Samples

This is a multicenter retrospective chart review of more than 1000 patients who underwent ThyroSeqV3 molecular testing at McGill University teaching hospitals between January 2016 to April 2022. All the adult patients (>18 years of age) with CNAs who had surgery were identified via our local database and were reviewed. Patients who were awaiting surgery or with an unavailable surgical pathology at the time of data collection were excluded. Molecular testing was done on FNA samples obtained from patients with atypia of undetermined significance or follicular lesion of undetermined significance (AUS/FLUS; Bethesda III) and follicular neoplasm/suspicious of follicular neoplasm (FN/SFN; Bethesda IV) to optimize the risk of cancer stratification, and on some of the patients with suspicious of malignancy (Bethesda V) and Bethesda VI results if it was expected that the result would change the extent of surgery. All included patients had a thyroidectomy (either lobectomy or total thyroidectomy), a sentinel lymph node biopsy, and a limited central neck dissection (CND). The decision on the extent of the surgery was made depending on the recommendations of American Thyroid Association (ATA) guidelines [21].

Data was collected on patient characteristics (including age and sex), nodule characteristics (including ultrasound findings, FNA results, molecular testing results, and pathological findings), and management decisions. The specific type and variants of the tumors were recorded as well. The research was approved by the Medical-Bioethics Research Ethics Committee (REC) of the integrated Health and Social Services Network for West-Central, Montreal (MP-05-2022-3177)

### 2.2. Tumor Analysis

#### 2.2.1. Cytologic and Histologic Diagnosis

Radiological evaluation with ultrasound imaging was done for all patients with thyroid nodules, and nodules were classified according to thyroid imaging, reporting and data system (TI-RADS) criteria [22]. Ultrasound-guided FNA (USFNA) was done by experienced thyroid specialists. The FNA (two passes) and the final pathology samples for all the included 67 nodules were evaluated by board-certified head and neck fellowship-trained pathologists. The Bethesda system for reporting thyroid cytology was used to report the diagnoses of the FNA samples [6]. The final pathological samples were diagnosed based on the 2017 World Health Organization (WHO) classification of endocrine tumors [23]. The pathology slides were re-examined, and the tumors were considered to be aggressive if one or more of the following features were present: macroscopic extrathyroidal extension (ETE), lymph node metastasis (LNM), poorly differentiated thyroid carcinoma (PDTC), and high-risk histological features (tall cell, columnar cell, hobnail/micropapillary, solid, and diffuse sclerosing).

#### 2.2.2. Molecular Analysis

After receiving their cytological diagnosis, all the management options, including molecular testing, were discussed with the patients depending on their clinical, radiological, and cytological findings according to the ATA guidelines for the management of thyroid nodules [21]. The rationale for performing molecular testing in patients with Bethesda III nodules and Bethesda IV nodules was to optimize the risk of cancer stratification and to avoid unnecessary surgeries. In patients with Bethesda V nodules and Bethesda VI nodules, it was utilized to determine the extent of the surgery required and if it was expected to be changed by the molecular testing result [21,24]. For those who decided to opt for molecular testing, another USFNA sample was taken and sent to a commercial laboratory at the University of Pittsburgh Medical Center (UPMC) for ThyroSeqV3 test to be analyzed for molecular alterations.

### 2.3. Statistical Analysis

A descriptive analysis was done. The samples were divided into two groups based on the postoperative pathology: malignant/NIFTP or benign. The malignant/NIFTP group was divided further into aggressive and non-aggressive groups. 

Statistical analyses of associations between the categorical variables were performed by the chi-square test or two-sided Fisher’s exact test and for the continuous variables with the non-parametric Mann–Whitney u test. The statistical significance between groups is identified using a threshold of *p*-value < 0.05. Logistic regression models were used to determine the most significant independent variables. Odds ratios are calculated. All analyses were performed using the statistical software package SPSS Version 28.

## 3. Results

### 3.1. Cohort Characteristics

Among the 316 surgically resected thyroid nodules that underwent molecular testing, 67 (21.2%) nodules were positive for CNAs. The mean age was 52.1 years (range: 27–81 years). Most of the patients were female (70.1%). Co-existing molecular alterations and mutations were found in 28 (41.8%) thyroid nodules. The baseline characteristics of the cohort are summarized in (Table 1). No statistically significant difference in age or sex was reported between patients with a CNA-positive benign nodule and those with a CNA-positive malignant/NIFTP nodule.

### 3.2. Tumor Characteristics

#### 3.2.1. Cytology

Most of the thyroid nodules with CNAs that underwent surgery were diagnosed as FN/SFN, with 31 of 67 (46.3%) thyroid nodules belonging to this category (Table 1). 23 nodules (34.3%) were diagnosed with AUS/FLUS, 12 (17.9%) were suspicious of malignancy (Bethesda V) and one nodule (1.5%) was cytologically malignant (Bethesda VI). No nodule was diagnosed as benign in cytology. 19 (61.29%) of the FN/SFN categorized nodules and 18 (78.26%) of the AUS/FLUS categorized nodules were non-oncocytic type.

#### 3.2.2. Molecular

**Figure 1:**
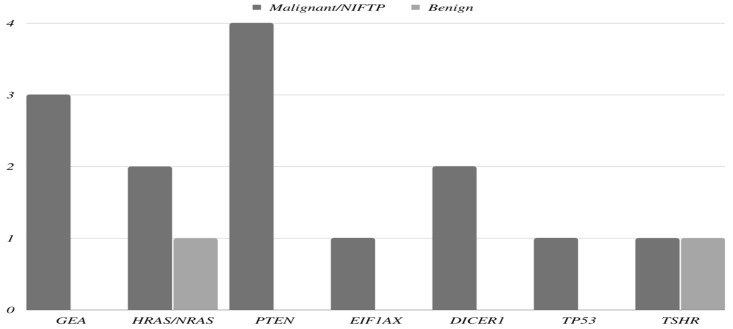
Prevalence of an additional molecular alteration based on tumor type.

**Figure 2:**
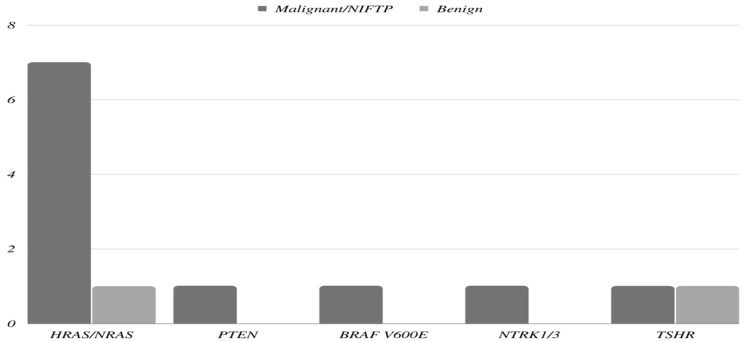
Prevalence of an additional molecular alteration and *GEA* based on tumor type.

CNAs were detected using ThyroSeq v3 molecular tests in all the thyroid nodules in our study. Co-existing molecular alterations and mutations were found in 28 (41.8%) thyroid nodules. The most common associated genetic alteration was *GEA*, which was found in 14 (20.9%) nodules. One nodule was associated with three molecular mutations (*EIF1AX, NRAS*, and *TP53*). Of the 14 nodules that were associated with concurrent *GEA* three had *GEA* alone and 11 had additional molecular alterations. All remaining 13 nodules were associated with one co-existing molecular alteration other than *GEA*. The molecular findings are summarized in Figure 1 and Figure 2. 

#### 3.2.3. Surgical Pathology

47 CNA-positive nodules were malignant/NIFTP (70.1%) (Table 1). Papillary thyroid carcinoma (PTC) was the most common detected malignancy (49.3%) with the follicular variant emerging as the most common variant (72.72%). Two nodules were diagnosed as PDTC, whilst another two had minor PDTC components. Six (9.0%) nodules were diagnosed as NIFTP on final pathology.

### 3.3. Patient Management 

Nine (13.4%) patients had a total thyroidectomy, and 58 (86.6%) had a hemithyroidectomy (Table 1). All the patients that had a total thyroidectomy had aggressive alterations or mutations on molecular testing, big nodule size (>4 cm), or contralateral nodule(s). Among those who had a hemithyroidectomy and were diagnosed with malignancy, seven (17%) had a completion thyroidectomy. Five of them had aggressive cancer, one had a tumor >4 cm, and one had a solid component on final pathology. One of the patients who did a completion thyroidectomy for aggressive cancer was booked initially for total thyroidectomy but had a hemithyroidectomy because there was a concern of possible recurrent laryngeal nerve neuropraxia. Ten patients received radioactive iodine (RAI) treatment postoperatively.

### 3.4. Factors That Indicate Malignancy and Aggressive Disease

Malignant tumors and NIFTPs were more common in females (74%) compared to males (60%) with positive CNAs. The mean age was 51.2 years for patients in the malignant/NIFTP group, and 54.4 years for patients in the benign group. No statistically significant difference in age or sex was found between the patients with positive CNAs that were diagnosed as malignant/NIFTP and those that were benign. On cytology, the Bethesda score was higher in the malignant/NIFTP group. The mean size was higher in the malignant/NIFTP group (2.65 cm) when compared to the benign group (2.13 cm); however, these findings were not statistically significant. The presence of other molecular alterations or mutations was associated with a higher risk of malignancy (*p* = 0.004) (Table 2) and aggressiveness (*p* = 0.033) (Table 3). Half of the patients with aggressive tumors were associated with more than one molecular alteration or mutation. However, the association was not statistically significant (*p* = 0.659), most likely due to the small sample size. Furthermore, all the aggressive co-existing molecular mutations or alterations (*BRAF V600E, TP53*, and *NTRK1/3* fusion) were associated with aggressive tumors. Out of the eight aggressive tumors, only one tumor was associated with metastatic LNs. The other seven tumors were associated with high-risk PTC subtypes or poorly differentiated components. The sensitivity and specificity of the presence of other molecular alterations with CNAs to predict malignancy were 53.2% and 85% respectively. The positive predictive value (PPV) was 89.3%, it means that 89.3% of the patients presenting a coexisting molecular alteration with CNA actually had thyroid cancer.

To investigate a predictive model for the risk of malignancy, we have used logistic regression models with age, gender, tumor size, Bethesda categories, tumor type and other genetic alterations as independent variables. In a univariate logistic regression model (Table 4), we observe that the presence of other molecular alterations or mutations is the only significant predictor for the risk of malignancy (OR = 6.439, 95% C.I. = 1.66–24.95, *p* = 0.007) with the presence of other molecular alterations or mutations having higher risk of malignancy. However, if we consider all independent predictors together in a multiple logistic regression model, we observe that tumor size (*p* = 0.032) is the most significant predictor with the risk of malignancy increasing with the tumor size. In this multiple logistic regression model, the presence of other molecular alterations or mutations still has higher risk of malignancy (OR = 5.087, 95% C.I. = 1.12–23.04, *p* = 0.035). This change in significance from the univariate to the multiple logistic regression model is mainly due to confounding of the tumor size and other variables. To predict the chance of aggressive tumors among the 47 patients with thyroid cancer (Table 5) we observe that none of the independent variables are significant for the risk of aggressiveness. To detect the significance or build a predictive model, we need more observations. The presence of other molecular alterations or mutations alterations have higher estimated risk aggressive tumors, but that is not significant at 5% level due to small number of the patients with aggressive tumors.

### 3.5. Tumor Type

Table 6 summarizes the tumor types of all nodules. CNA-positive tumors ended up being follicular patterned tumors in 41 (65.2%) thyroid nodules, oncocytic patterned tumors in 20 (29.9%) thyroid nodules and other tumors which includes PTC, PDTC, and benign tumors in 6 (8.9%) thyroid nodules.

## 4. Discussion

In our study, the prevalence of CNAs in thyroid nodules that underwent molecular testing followed by surgical resection is high (21.1%). This finding is comparable to the ThyroSeq v3 validation study (21%) and TGCA cohort (27%) findings [11,25]. Thyroid cancer was found in 70.1% of thyroid nodules with CNAs in our study compared to 59% in the ThyroSeq v3 validation study [25]. However, our study population included Bethesda III, IV, V and VI thyroid nodules, when compared to the ThyroSeq v3 validation study that studied Bethesda III and IV categories only, and the TGCA cohort that studied PTC exclusively. The presence of other molecular alterations and mutations was found to significantly increase the risk of malignancy to 89.3%, compared to 56.4% in nodules with isolated CNAs. This association was still statistically significant in univariate and multivariate analysis. The high odds ratio (5.09) and the wide CI (1.12–23.04) in multivariate analysis can be related to the variable genomic expression of the different associated molecular alterations and mutations in the nodules with the CNAs. Furthermore, in multivariate analysis, tumor size becomes a significant predictor for the risk of malignancy.

Aggressive tumors were found in eight (11.9%) thyroid nodules with CNAs that underwent surgery. Seven of them were associated with other molecular alterations, four were associated with high-risk mutations or alterations (*BRAF V600E, TP53*, and *NTRK1/3* fusion), two were associated with *PTEN* mutation with high AF (70% and 90%), and one was associated with *GEA* only. The presence of other molecular alterations and mutations elevated the risk of aggressiveness from 17.02% to 29.2%. Furthermore, a correlation between the number of associated molecular alterations and mutations and aggressiveness could not be established. 97.4% of the isolated CNA-positive nodules and all CNA-positive nodules that were associated with *RAS* mutations (*NRAS* and *HRAS*) or other low-risk alterations like *DICER1,* and *EIFAX1* alone or with *GEA* were either benign or non-aggressive malignant tumors/NFTIPs that could have benefitted from a lobectomy alone. CNA-positive thyroid nodules that were associated with high-risk mutations like (*BRAF V600E, TP53*, and *NTRK1/3* fusion) or *PTEN* with high AF might benefit from more aggressive treatment like a total thyroidectomy +/− CND +/− RAI treatment.

In our study, PTC was the most common tumor type associated with CNA-positive thyroid nodules (49.3%). Other tumors that were associated with CNAs were NIFTPs (8.9%), oncocytic carcinomas (OC) (6%), follicular thyroid carcinomas (FTCs) (3%), PDTCs (3%) and benign tumors (29.9%). In a study conducted by W D Doerfler et al., 60.3% of thyroid nodules with CNAs ended up being oncocytic tumors (24 oncocytic carcinomas and 43 oncocytic adenomas) [26]. In our study, 28.4% were oncocytic tumors including OC, oncocytic PTC, oncocytic adenomas, oncocytic metaplasia, and PDTC arising from OC. 62.7% of nodules were follicular lesions such as the follicular variant of PTC (FVPTC), NFTIP, FTC, and follicular adenoma. Lastly, 8.9% were other tumors including tall cell PTC, classic PTC, solid/trabecular PTC, PDTC, and benign. The histopathological diagnoses for all nodules are summarized in Table 6. McKelvey et al. showed a strong association between *TERT* and *BRAF* CNAs and *RAS*-like tumors especially FVPTC [27]. The findings of both studies (W D Doerfler et al. and McKelvey et al.) are compatible with our results: most of the thyroid nodules with CNAs were either follicular patterned tumors or oncocytic tumors. While it is not yet clear when CNAs can cause oncocytic tumors or follicular patterned tumors, Gopel R et al. presented widespread chromosomal loss near haploid state as a driver alteration for OC [28]. Another study showed that follicular adenomas have a high frequency of CNAs, specifically, amplifications of chromosomes 7 and 12 [29]. Further studies are needed to explore the factors that might participate in developing either follicular or oncocytic tumors in CNA-positive thyroid nodules. It is worth mentioning that in our study, oncocytic cells were mentioned in the cytology of 68.4% of the oncocytic tumors and 9.8% of the follicular lesions. It is well known, however, that oncocytic changes are related to the accumulation of mitochondria in the cytoplasm, representing a spectrum, and that oncocytic neoplasms are arbitrarily defined by consisting of >75% oncocytic cells according to WHO.

Multiple studies explored the association of the thyroid nodule with the nodule size as a predictor of malignancy according to the different thyroid tumor types [30,31,32,33]. In those studies, the nodule size was positively related to the risk of malignancy in the nodules with FTCs, FVPTCs and OC, in contrast to the nodules with PTCs which the nodule size is usually inversely related to the risk of malignancy. In our study, the positive relationship of the size of the nodule with the risk of malignancy that was found in the multivariate analysis can be explained to be more likely related to the fact that most of the tumors that arise from the CNAs-positive nodules are either follicular patterned tumors or oncocytic tumors.

The understanding of molecular markers in thyroid nodules progressed dramatically in the last decade. This wealth of knowledge has been translated into clinical application with the help of molecular testing. Currently, molecular tests can provide diagnostic, prognostic, and therapeutic information that can influence the management and decision-making of patients with thyroid nodules [10]. In the TCGA cohort, CNAs have been identified as a driver molecular alteration for thyroid cancer [11]. In addition, CNAs can act as a promoter of other alterations by changing the gene expression of the affected gene [20]. The findings of our study confirm that CNAs alone can be a driver molecular alteration for thyroid tumorigenesis. Furthermore, the presence of co-existing molecular alterations with CNAs is associated with a higher risk of malignancy, suggesting that CNAs might play a role in promoting the effect of other molecular alterations. Our study failed to identify any significant association between CNA-positive thyroid nodules and aggressiveness due to the small sample size. Further studies with a larger sample size are necessary to discover the factors that can be related to aggressiveness. However, in concurrence with the literature, our study’s findings explicate that an increase in mutational burden (presence of other molecular alteration or mutations) is associated with a higher risk of malignancy and aggressiveness [10]. In addition, most of the CNA-positive thyroid nodules are either follicular or oncocytic tumors. Our study presented the prevalence of CNAs in thyroid nodules that underwent molecular testing followed by surgery and provided clinical, cytological, and pathological characteristics of these nodules. We believe that the findings of our study can impact clinical decision-making of patients with CNA-positive thyroid nodules and can be a valuable bedrock for future research.

There are several limitations in this study, including the inherent limitations of a retrospective study. As a single-center study taking place in the urban city of Montreal, Canada, a geographic selection bias was introduced. Additionally, the study is subject to another selection bias since molecular testing for CNAs performed was paid for by the patient. Therefore, not all patients that potentially had CNAs underwent molecular testing. As a result, malignant tumors that harbor CNAs may be underrepresented in this study. Future studies should involve more comprehensive molecular testing and a multicenter approach to minimize these limitations.

## 5. Conclusions

In our study, most of the thyroid nodules with CNAs that underwent surgery were malignant. The presence of other molecular alterations and larger tumor size increased the risk of malignancy. Thyroid nodules with CNAs and high-risk alterations or *PTEN* mutation with high AF were associated with aggressive cancers. Thyroid nodules with CNAs alone or in conjunction with lower risk mutations such as *HRAS, NRAS, DICER1, EIF1AX*, and *GEA* are more likely to be low-risk neoplasms. Most CNA-positive thyroid nodules were either oncocytic tumors or follicular patterned tumors. Further studies are needed to explore factors that are related to aggressiveness and the development of each type of tumors.

## Figures and Tables

**Table 1:** Clinical and pathological characteristics of the 67 patients with thyroid cancer included in this study.

Variant	Population *n* (%)
Age (years) mean (min-max, ± SD)	52.15 (19–81 ± 14.113)
Sex Female Male	47 (70.1) 20 (29.9)
Dominant nodule size (cm) mean (min-max, ± SD)	2.494 (0.7–8 ± 1.394)
Bethesda category	
Bethesda 3	23 (34.3)
Bethesda 4	31 (46.3)
Bethesda 5	12 (17.9)
Bethesda 6	1 (1.5)
Molecular profile testing	
Other genetic alterations/mutations	28 (41.8)
No other genetic alterations/mutations	39 (58.2)
Thyroidectomy	
Hemi thyroidectomy	51 (76.1)
Total thyroidectomy	9 (13.4)
Completion thyroidectomy	7 (10.4)
Histopathology	
Benign	20 (29.9)
Malignant/NIFTP	47 (70.1)
Papillary thyroid cancer	33 (70.2)
Follicular thyroid cancer	2 (4.3)
Oncocytic thyroid cancer	4 (8.5)
PDTC	2 (4.3)
NIFTP	6 (12.7)

PDTC: Poorly differentiated thyroid cancer, NIFTP: non-invasive follicular neoplasm with papillary like features.

**Table 2:** Association between malignancy and clinicopathological characteristics in the 67 patients with CNA-positive thyroid nodules that underwent surgery.

Variant	Malignant/NIFTP *n* (%) (*n* = 47)	Benign *n* (%) (*n* = 20)	*p*-Value
Age (years) mean (min-max, ± SD)	51.21 (19–79 ± 14.093)	54.35 (29–81 ± 14.276)	0.409
Sex			
Female	35 (74.5)	12 (25.5)	0.236
Male	12 (60.0)	8 (40.0)	
Tumor size (cm) mean (min-max, ± SD)	2.65 (0.8–7.5 ± 1.2664)	2.13 (0.7–8 ± 1.633)	0.159
Bethesda category			0.124
Bethesda 3	12 (52.2)	11 (47.8)	
Bethesda 4	24 (77.4)	7 (22.6)	
Bethesda 5	10 (83.3)	2 (16.7)	
Bethesda 6	1 (100.0)	0 (0)	
Molecular profile			
Other molecular alterations/mutation	25 (89.3)	3 (10.7)	0.004
No Other molecular alteration/mutation	22 (56.4)	17 (43.6)	
Tumor Type			0.195
Oncocytic	11 (55.0)	9 (45.0)	
Follicular	31 (75.6)	10 (24.4)	
Other	5 (83.3)	1 (16.7)	

**Table 3:** Association between aggressive tumors and clinicopathological characteristics in the 47 patients with thyroid cancer.

Variant	Aggressive Tumors *n* (%) (*n* = 8)	Non-Aggressive *n* (%) (*n* = 39)	*p*-Value
Age (years) mean (min-max, ± SD)	47.5 (25–79)	51.9 (19–75)	0.400
Sex			0.394
Female	5 (14.3)	30 (85.7)	
Male	3 (25.0)	9 (75.0)	
Tumor size (cm) mean (min-max, ± SD)	3.11 ± 2.05	2.55 ± 1.05	0.711
Bethesda category			0.156
Bethesda 3	2 (16.7)	10 (83.3)	
Bethesda 4	4 (16.7)	20 (83.3)	
Bethesda 5	1 (10.0)	9 (90.0)	
Bethesda 6	1 (100.0)	0 (0.0)	
Molecular profile			0.033
Other molecular alterations/mutations	7 (28.0)	18 (72.0)	
>1 molecular	4 (33.3)	8 (66.7)	0.659
One molecular	3 (23.1)	10 (76.9)	
No Other molecular alteration/mutation	1 (4.5)	21 (95.5)	
Tumor Type			0.144
Oncocytic	3 (27.3)	8 (72.7)	
Follicular	3 (9.7)	28 (80.3)	
Other	2 (40.0)	3 (60.0)	

**Table 4:** Logistic regression results to predict the chance of malignancy using clinicopathological characteristics in the 67 patients with CNA-positive thyroid nodules that underwent surgery.

Variant	Univariate			Multivariate		
	OR	95% C.I.	*p*-Value	OR	95% C.I.	*p*-Value
Gender: Male	0.514	0.17–1.56	0.240	0.259	0.06–1.24	0.090
Gender: Female	1.000	-	-	1.000	-	-
Age	0.984	0.95–1.02	0.404	1.003	0.95–1.06	0.913
Tumor Size	1.401	0.87–2.25	0.163	1.909	1.06–3.43	0.030
Bethesda 3	0.218	0.04–1.22	0.084	0.148	0.02–1.03	0.053
Bethesda 4	0.686	0.12–3.89	0.686	0.541	0.08–3.48	0.518
Bethesda 5	1.000			1.000	-	-
Bethesda 6	-	-	-	-	-	-
Other Genetic Alteration	6.439	1.66–24.95	0.007	5.087	1.12–23.04	0.035

**Table 5:** Logistic regression results to predict the chance of aggressive tumors using clinicopathological characteristics in the 47 patients with thyroid cancer.

Variant	Univariate			Multivariate		
	OR	95% C.I.	*p*-Value	OR	95% C.I.	*p*-Value
Gender: Male	2.000	0.40–10.04	0.400	4.017	0.39–41.65	0.244
Gender: Female	1.000	-	-	1.000	-	-
Age	0.977	0.93–1.03	0.412	0.951	0.88–1.02	0.178
Tumor Size	1.378	0.78–2.43	0.269	1.657	0.74–3.72	0.221
Bethesda 3	1.800	0.14–23.37	0.653	1.753	0.07–43.76	0.732
Bethesda 4	1.800	0.18–18.47	0.621	2.371	0.13–44.49	0.564
Bethesda 5	1.000			1.000	-	-
Bethesda 6	-	-	-	-	-	-
Other Genetic Alteration	8.17	0.92–72.81	0.060	6.200	0.60–64.46	0.127

**Table 6:** Tumor types according to the final histopathology of all the 67 CNA-positive thyroid nodules.

Tumor Types		
OT (*n* = 19)	FT (*n* = 42)	Other (*n* = 6)
Benign (*n* = 9)	Benign (*n* = 10)	Classic PTC (*n* = 2)
HCC (*n* = 4)	FVPTC (*n* = 24)	Tall cell PTC (*n* = 1)
Oncocytic PTCs (*n* = 5)	NIFTP (*n* = 6)	Solid/trabecular PTC (*n* = 1)
PDTC (*n* = 1)	FTC (*n* = 2)	PDTC (*n* = 1)
		Benign (*n* = 1)

OT: Oncocytic tumors, FT: Follicular tumors, PDTC: Poorly differentiated thyroid cancer, FVPTC: follicular variant papillary thyroid cancer, NIFTP: non-invasive follicular neoplasm with papillary like features, FTC: Follicular thyroid cancer.

## Data Availability

The data presented in this study are available on request from the corresponding author. The data are not publicly available due to ethics approval agreement.
